# The Role of Tubulin Polymerization-Promoting Protein2 (TPPP2) in Spermatogenesis: A Narrative Review

**DOI:** 10.3390/ijms25137017

**Published:** 2024-06-27

**Authors:** Ferenc Orosz

**Affiliations:** Institute of Molecular Life Sciences, Research Centre for Natural Sciences, HUN-REN, 1117 Budapest, Hungary; orosz.ferenc@ttk.hu

**Keywords:** epididymis, flagellum, infertility, oligoasthenozoospermia, sperm, spermatozoa, spermiogenesis, testis

## Abstract

Tubulin polymerization-promoting protein2 (TPPP2) is one of the three paralogs of mammalian TPPP proteins. Its possible role in spermatogenesis is described in this narrative review. TPPP2 is expressed specifically in the male reproductive system, mainly in testes and sperm, and also in the epididymis. In testes, TPPP2 is exclusively expressed in elongating spermatids; in the epididymis, it is located in the middle piece of the sperm tail. TPPP2 is involved in spermiogenesis, in steps which are determinative for the formation and morphology of spermatids. The inhibition of TPPP2 decreases sperm motility (the curvilinear velocity of sperms), probably due to influencing mitochondrial energy production since TPPP2 knockout mice possess an impaired mitochondrial structure. There are data on the role of TPPP2 in various mammalian species: human, mouse, swine, and various ruminants; there is a significant homology among TPPP2s from different species. Experiments with *Tppp2*^−/−^-mice show that the absence of TPPP2 results in decreased sperm count and serious dysfunction of sperm, including decreased motility; however, the in vitro capacitation and acrosome reaction are not influenced. The symptoms show that *Tppp2*^−/−^-mice may be considered as a model for oligoasthenozoospermia.

## 1. Introduction

The aim of this narrative review is to describe the possible role of the tubulin polymerization-promoting protein2 (TPPP2) in spermatogenesis. Based on its specific occurrence (male genitalia-cf. Section 3), it is a rational assumption that this protein may have some role in this complex process. The PubMed database was searched using TPPP2 as query for publications. Fourteen papers were found and retrieved (Accessed 2 May 2024), ten of which were relevant to spermatogenesis and/or the male reproductive system. These are references [1,2,3,4,5,6,7,8,9,10] (Table 1). The other articles were mostly the author’s evolutionary and phylogenetic works.

## 2. The TPPP Protein Family

The namesake of the TPPP protein family is the tubulin polymerization-promoting protein (TPPP/p25 or TPPP1) [11,12]. The name indicates its tubulin polymerizing and microtubule bundling properties. There are three members of the family in mammals and other vertebrates: TPPP1, TPPP2, and TPPP3 [13]. The human paralogs display about 60% identity with each other [13]. They may have arisen at the dawn of vertebrate evolution by a two-round whole-genome duplication [14]. It was suggested that the single non-vertebrate *tppp* gene was duplicated in the first round of whole-genome duplication in the vertebrate lineage giving rise to *tppp1* and the precursor of *tppp2*/*tppp3* [15,16]. TPPP orthologs are also present in some protists and algae [17]. A strong correlation was suggested between the incidence of *tppp*/TPPP genes/proteins and that of the eukaryotic flagellum [18,19].

TPPP1 is brain-specific; it has an important role in oligodendrocyte differentiation and myelinization [20]. In synucleinopathies (e.g., Parkinson’s disease, multiple system atrophy), it is abnormally enriched in inclusion bodies [21,22]. The occurrence of TPPP3 is ubiquitous; it is involved in developmental processes of the musculoskeletal system [23,24]. Pathologically, higher mRNA and/or protein expression of TPPP3 was found in tumor cells in comparison to normal ones in most cases investigated up to now, mostly in various lung tumors (reviewed in Ref. [25]).

## 3. TPPP2

For a long time, we had much less knowledge about TPPP2. It was first found in 2002 and was cloned from a human fetal brain cDNA library; however, it has not been detected in adult mammalian brains [26]. The *TPPP2* gene is located on chromosome 14 [26]. Our preliminary studies concerning its properties revealed that in contrast to TPPP1 and TPPP3, which are strongly linked to microtubules, TPPP2 distributes homogeneously within the cytosol of transfected HeLa cells [13]. Its affinity towards tubulin and microtubules was found to be an order of magnitude weaker than those of the other two paralogs [13].

**Figure 1:**
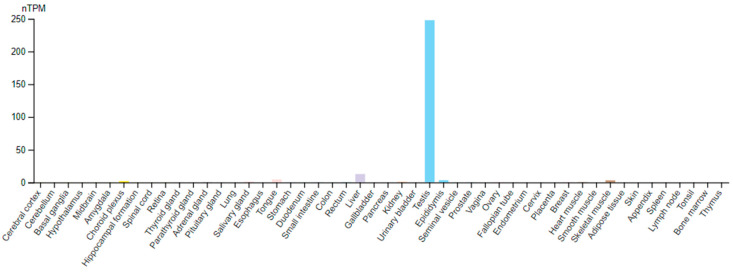
TPPP2 RNA expression data in normal human tissues combining the Genotype Tissue Expression (GTEx) and Human Protein Atlas (HPA) transcriptomics datasets. (https://www.proteinatlas.org/ENSG00000179636-TPPP2/tissue) [27] (accessed on 18 March 2024). nTPM: normalized protein-coding transcripts per million.

Today it can be said that its occurrence is largely linked to the testis (Figure 1). Protein expression data based on immunohistochemistry also strengthen that TPPP2 is by far the most abundant in testis, although its presence in some other tissues (nasopharynx, bronchus, fallopian tube, endometrium) was also suggested (Human Protein Atlas-HPA [27]). The discrepancy is caused very probably by the cross-reactivity of the antibody used. (According to the HPA home page, the applied antibody recognized, in addition to human TPPP2, also mouse TPPP1, TPPP2, and TPPP3 at 59%, 65% and 85%, respectively. The author had similar experiences when examining the cross-reactivity of antibodies with human TPPPs (unpublished). In fact, all of the aforementioned tissues are characterized by very high levels of TPPP3 according to HPA.) Another male reproductive organ, the epididymis, also expresses TPPP2, but in a much less (about two orders of magnitude lower) amount.

There are other examples when one of the paralogs of a gene/protein is expressed specifically in sperm and testes. For example, the house-keeping glycolytic enzyme, glyceraldehyde 3-phosphate dehydrogenase-2 gene (*GAPDHS*), is expressed only in spermatogenic cells [28].

## 4. TPPPs and Flagellum

TPPPs occur predominantly in species that are flagellated. The eukaryotic flagellum is a microtubule-based organelle; thus, the connection is self-evident. The correlation between the occurrence of a flagellum and TPPPs, based on bioinformatic analysis of genomic and proteomic data, is strong: in general, with very few exceptions, flagellated organisms do contain TPPP proteins; on the other hand, in non-flagellated species, TPPPs usually do not occur [18,19]. Thus, TPPP is absent in prokaryotes and archaea, as well as in terrestrial plants. However, there is little evidence for the functional relationship between TPPPs and the flagellum. The connection was first shown in *Chlamydomonas reinhardtii*, a biflagellate green alga [29]. Its TPPP ortholog, the Flagellar Associated Protein 265 (FAP265 protein), can be found in the flagella, and is inevitable in their formation, as demonstrated by using FAP265 null mutants [29]. Another example came from the parasitic phylum, Apicomplexa. In many apicomplexans, flagella are found in male microgametes. In the *Plasmodium* genus, *P. falciparum* TPPP (PFL1770c; XP_001350760) was shown to be essential for gametocytogenesis, using piggyBac transposon-mediated insertional mutagenesis, but the mutant failed to form mature gametocytes [30]. In another species of this genus, *P. yoelli,* it has also been found that TPPP (*Py*05543; EAA17578) is necessary for male gametocyte formation: *Py*05543 knockout (KO) parasites, obtained by CRISPR/Cas9-mediated genome editing, were deficient in this respect [31].

The assembly of the flagellum as a motility apparatus is one of the key steps of human spermatozoa formation [32,33]; thus, the occurrence of a TPPP paralog, namely TPPP2, in testis and sperm is not surprising. Taking into account its tissue-specific expression, it suggests that TPPP2 might be involved somehow in the process of spermatogenesis.

## 5. Flagellum, Spermatogenesis, and Its Defects

Male germ cells are produced during spermatogenesis, which takes place in the testis. Spermatogenesis is defined as the differentiation of spermatogonial stem cells into spermatocytes and the production of spermatids from them [32]. Then, spermatids are transformed to spermatozoa in the final phase of spermatogenesis. This final phase of sperm development, called spermiogenesis, involves spermatid differentiation, which includes the condensation and elongation of the nucleus, the biogenesis of the acrosome developed from the Golgi complex, and the assembly of the flagellum [32,33]. The spermatozoa produced have to transit through the epididymis to reach maturity and gain their forward motility and ability to meet the egg [34]. The final step is capacitation when the sperm stays in female genital tracts and spermatozoon becomes able to fertilize [35]. As a part of capacitation, sperm develops an asymmetrical flagellar beating known as hyperactivation and acquires the ability to undergo the acrosome reaction [36].

The motility of the spermatozoon is provided by its flagellum. Sperm flagellum shares its structure with other eukaryotic flagella: their main constituent is the axoneme, consisting of nine doublet microtubules surrounding two central single microtubules. The axonemal microtubules nucleate and extend from a basal body, a centriolar structure [37]. The three parts of human sperm flagella are the mid(dle) piece, principal piece, and end-piece [38]. The mid- and principal pieces surround the axoneme, and outer dense fibers are present in both of them. The mitochondria are arranged helically to the middle piece; a fibrous sheath exists in the principal piece. No peri-axonemal structures can be found in the end-piece [39]. Defects in genes involved in these processes result in the defects in the counts, motility and morphology of sperms—oligozoospermia, asthenozoospermia, teratozoospermia, respectively—and their various combinations (oligo-astheno-teratozoospermia) thus contribute to male infertility.

## 6. Early Proteomic Data

Proteomics is the systematic study of the occurrence of proteins in a given cell, tissue or organ. Proteomic studies of various organs significantly increased the knowledge of the distribution and role of proteins. For a long time, only a few proteins in the human testis were identified due to limitations of available technology [40]. The first attempt to organize and catalog the human spermatozoa proteome was performed in 2011 [41]. The first proteomic data showing that TPPP2 is present in testes and seminal fluid were published only a decade ago [1,2].

Liu et al. [1] identified 7346 proteins within the human testis, using 2D HPLC–tandem mass spectrometry (HPLC-MS/MS) analysis, with a high confidence. Based on these results, a human testis and sperm proteome database was constructed, giving an overall view of the testis and sperm proteins. Plenty of the identified testis proteins were found to be associated with infertility. Liu and colleagues’ results also provided a molecular connection between spermatogenesis and tumorigenesis, and they characterized several new cancer/testis genes. Besides *TPPP2*, the other genes were *DMRT1*, *HEMGN*, *PIWIL1*, *PRSS55*, and *TMPRSS12*. (There are corresponding features between cancer cells and those in the germ cell differentiation pathways [42].) The potential medical importance of the proteins encoded by these genes is that because they are not expressed in somatic cells, they are often recognized by the immune system of cancer patients and might therefore be used as tumor vaccines [42]. Although this connection is an interesting phenomenon, it is out of the scope of the present review.

Interestingly, further data concerning the testicular occurrence of TPPP2 came from the proteomic studies of ruminants. In the same year, Martins et al. [2] identified the major heparin-binding proteins in the seminal plasma from reproductively sound Morada Nova rams. The heparin affinity chromatography was followed by one-dimensional SDS-PAGE separation, and the digested bands were analyzed by tandem mass spectrometry. Only one-sixth of the seminal plasma proteins was found to bind to heparin. The authors suggested that heparin-binding proteins of the ram seminal plasma affect several features of the reproductive processes, such as sperm capacitation, sperm–egg binding, the formation of the oviduct reservoir, and protection against microorganisms and oxidative stress [2]. The method used allowed the identification of proteins never reported before in ram seminal plasma, including TPPP2.

## 7. Proteomic Data from Ruminants

Further proteomic data were obtained from other ruminants, such as buffalo, yak, and bull. The reason for the investigation of this livestock’s sperm is that artificial insemination has a significant role in the breeding of these animals; thus, knowing the sperm fertilizing potential is an important issue. Differences in the protein composition between the spermatozoa of male animals with high and low fertility can be used for fertility prediction.

Aslam and co-workers compared the sperm proteome of buffalo (*Bubalus bubalis*) bulls with high and low fertility by applying two-dimensional difference gel electrophoresis combined with mass spectrometry [3]. In high-fertility animals, 10 and 15 proteins were overexpressed and underexpressed at the level of twofold or more, respectively. The proteins overexpressed in low-fertility spermatozoa included TPPP2 as well (2.2-fold overexpression). The other proteins of this type were ATP5F1, CS, CTSL1, EGFL6, GPR4, HARS, IDH3A, MT1A, PMP2, PRODH2, SRPK3, TCRB, TUBB2B, and Uncharacterized protein C9orf9 homolog isoform X4. Most of these proteins were connected to energy metabolism, capacitation, acrosome reaction, and cryopreservation. This knowledge is especially important in the case of buffalo since its sperm is very sensitive to cryopreservation [43].

Cryopreservation can influence the effectivity of sperm in general, not only in the case of buffalo; frozen-thawed semen may have less favorable properties due to various side effects such as decreased sperm motility, mitochondrial dysfunction, elevated levels of reactive oxygen species, and damages in cell membranes [44]. Zhang et al. [4] analyzed, using tandem mass tag labeling combined with liquid chromatography–tandem mass spectrometry (LC-MS/MS), the phosphoproteome of semen originated from yak (*Bos grunniens*) and treated in different ways: frozen-thawed high-motility sperm, sperm incubated in artificial capacitation medium, and sperm of acrosome reaction (inducted by a calcium ionophore, A23187). The phosphoproteomes obtained in different conditions were compared. TPPP2 was among the 220 proteins which were down-regulated in sperms of acrosome reaction conditions compared to frozen-thawed high-motility sperms. The authors concluded that TPPP2 plays a role in sperm motility; indeed, this has been explicitly demonstrated by others (see Section 8 and Section 9). However, considering the phosphorylation sites of TPPP2, both up- and down-regulation occurred. Protein–protein interaction analysis of phosphorylated proteins using bioinformatic methods suggested that differentially phosphorylated proteins (among them, TPPP2) related to capacitation and acrosome reaction process played essential roles in various processes such as sperm motility. Among the interacting partners of TPPP2, GAPDHS (glyceraldehyde 3-phosphate dehydrogenase-2), SSMEM1, TEX33, TSSK4 and TRIM42 were identified. It is interesting to note that the brain-specific TPPP paralog (TPPP1 or TPPP/p25) is also a phosphoprotein, and the phosphorylation status of its phosphorylation sites influences its properties and interactions [45,46]. Additionally, TPPP1 was also shown to interact with the somatic glyceraldehyde 3-phosphate dehydrogenase paralog, GAPDH-1 [47].

Gacem et al. [5] compared the sperm proteome of bulls (*Bos taurus*) with high and low fertility. They used sequential window acquisition of all theoretical mass spectra (SWATH-MS) analyses and compared several in vitro parameters such as sperm motility, sperm viability, mitochondrial membrane potential, and reactive oxygen species of spermatozoa. There were significant differences between the properties and the protein profile of the high and low fertility group. For example, not surprisingly, the motility of spermatozoa of high-fertility bulls was about 40% higher than that of the low-fertility ones. The proteins which were more abundant in the former group were mostly related to sperm motility (TPPP2, SSMEM1, SPAG16) and to energy production (COX7C). In contrast to the results obtained in [3], in this study, TPPP2 was moderately overrepresented in the high fertility group.

## 8. Bioinformatic Analyses

Gòdia and co-workers [6] analyzed 25 sperm-related phenotypes (e.g., sperm concentration, percentage of morphologically abnormal sperm cells, various sperm motility traits as the percentage of motile spermatozoa, percentage of viable cells, percentage of morphologically abnormal acrosomes, and average curvilinear and straight-line velocities) in swine (*Sus scrofa*) and their connection with genetic and transcriptomic data, using multiple bioinformatics tools. TPPP2 was found to be the fifth most abundant mRNA in sperm, suggesting that it is a candidate gene influencing sperm characteristics and fertility. (The other most abundant protein-coding transcripts associated with sperm functions were DNAJB8, OAZ3, PRM1, and TNP1). The connection between the phenotypes and the abundance of mRNAs was investigated; they found 6128 significant correlations involving 3007 genes. In the case of *Tppp2*, a highly positive correlation was established with curvilinear velocity of sperms freshly collected and measured after 5 min of collection.

Recently, similar bioinformatic analyses have been used by several groups to identify the key genes associated with non-obstructive azoospermia (failure of spermatogenesis, leading to the absence of spermatozoa in ejaculates) using human testis microarray expression profile datasets available at the NCBI’s Gene Expression Omnibus (GEO) database [7,48,49]. The datasets used were the same or overlapped with each other in the different studies. The authors analyzed the differences between normal and infertility datasets and identified the differentially expressed genes connected to azoospermia. A protein–protein interaction network was constructed, and the hub genes were identified. Interestingly, the three groups identified different genes as the most important ones, probably because the datasets were only partially overlapped (two of them, datasets GSE45885 and GSE45887, were common from the 2, 3, and 4 datasets used by the various groups). One of the research groups found *TPPP2* among the nine hub genes (the further genes were *TEX38*, *FAM71F*, *PRR30*, *FAM166A*, *LYZL6*, *ARMC12*, *SPACA4*, and *FAM205A*), regulated by protein kinases, of the interaction network [7]. The role of protein kinases emphasizes the importance of phosphorylation in the regulation of processes related to spermatogenesis, as demonstrated in Ref. [4] (cf. Section 7). TPPP2 was about 10-fold up-regulated in the non-obstructive azoospermia (NOA) patients compared to normal spermatogenesis persons.

## 9. Knockout Mice Models of Oligoasthenozoospermia and Asthenoteratozoospermia TPPP2 Is Involved in Spermiogenesis

The work of Zhu et al. [8] contributed the most to the understanding of the physiological/pathological role of TPPP2. They studied TPPP2 KO mice as well as human and mouse samples to enlighten its function. The expression profile of mouse TPPP2 was analyzed using RT-PCR and immunohistochemistry. TPPP2 was found specifically in male reproductive organs, the testes and epididymis. In the testes, TPPP2 was exclusively expressed in elongating spermatids at stages IV-VIII of the seminiferous epithelial cycle that correspond to the spermatids at steps 15–16 of spermiogenesis, which are determinative for the formation and morphology of spermatids. In the epididymis, TPPP2 was expressed in sperm and was localized in the middle piece of the sperm tail.

Further results showed that the inhibition of TPPP2 hindered sperm motility. Human or mouse sperm were co-incubated with anti-TPPP2 antibodies, and 20 μg/mL anti-TPPP2 antibody significantly reduced the sperm motility. Moreover, the sperm ATP content in the antibody-treated samples was significantly lower than that in the controls, which indicates that the two phenomena may be related to each other. Additionally, the in vitro fertilization rate was also significantly lower than that in the control treated with IgG antibody instead of anti-TPPP2 antibody. However, the in vitro capacitation and acrosome reaction were not influenced.

*Tppp2*^−/−^-mice were established where testes and sperm did not express TPPP2 protein. The sperm counts and sperm motility of KO mice were much lower than those of the controls. Morphologically, the KO and control mice were rather similar. No difference in the size and weight of the testes occurred. Both groups possessed intact spermatogenic tubules and cells of normal shape. Even the number of spermatocytes was similar. Thus, the *Tppp2*^−/−^-mice can be considered as a model for oligoasthenozoospermia.

The reason for the decreased sperm count and motility was attributed to mitochondrial dysfunctions. In KO mice, a significant proportion of sperm lacked inner mitochondrial membrane cristae. The mitochondrial membrane potential in mutant mice sperm was lower than in the sperm of wild-type mice. Components of the complexes of electron transfer chain, located in the inner mitochondrial membrane, showed reduced expression compared with those of the control mice. In connection with this, the ATP content was also lower. Since the decrease in the mitochondrial membrane potential can be related to apoptosis, not surprisingly, an increased apoptotic index was also observed in the sperm of KO animals.

The mitochondria are arranged helically to the middle piece of the sperm tail where TPPP2 is located [8,50]; thus, this connection between the mitochondrial defects and the absence of TPPP2 can be understood. Finally, the group found that TPPP2 is localized in the cytosol and is not aligned along the microtubules in accordance with our former results [13]. The *Tppp2*^−/−^-mice sperm tails contained normal microtubules. These facts indicate that the properties of TPPP2 differ from its paralogs, TPPP1 and TPPP3, that interact with microtubules, as we had shown [11,51].

A mouse model of asthenoteratozoospermia (defects in the motility and morphology of sperm) was established by Wang and his co-workers [9,50]. The mice were knocked out for *Cfap65* (cilia- and flagella-associated protein 65) gene. It was shown that mutations of this gene resulted in acrosome hypoplasia and flagellum malformations in humans, causing serious asthenoteratozoospermia [50]. It is in accordance with the fact that CFAP65 was shown to be localized at both the acrosomal region and the flagellar mid-piece of spermatozoa in humans [50]. The importance of CFAP65 in sperm head shaping, acrosome formation, and flagellar assembly was demonstrated by the authors [9].

Knocking out CFAP65 significantly influenced the level of 182 proteins as shown by reverse phase HPLC–mass spectrometer/mass spectrometer-based proteomic analysis [9]. A comparison of the data between the KO and the wild-type mice revealed that 59 and 123 proteins were significantly up- and down-regulated, respectively. TPPP2 was among the several mitochondrial function-related proteins whose levels decreased [9]. Another such protein was NSUN4 (NOP2/Sun RNA methyltransferase 4). Both proteins have recently been proposed to be hepatocellular carcinoma biomarkers, i.e., prognostic indicators of this tumor (TPPP2 in Ref. [10]; NSUN4 in Ref. [52]). Thus, NSUN4, similarly to TPPP2, can also be considered as a cancer/testis protein (cf. Ref. [1] in Section 6).

Immunoprecipitation and immunostaining experiments with testes of wild-type mice revealed that CFAP65 probably forms a protein network containing TPPP2 as well as MNS1, RSPH1, ZPBP1, and SPACA1. It is worth noting that both CFAP65 and TPPP2 can be found in the middle piece of the human mature sperm tail. Based on all these results and taking into consideration the mitochondrial role of TPPP2 in sperm flagellum shown in [6], the authors hypothesized that TPPP2 might be a partner of CFAP65 during mitochondrial sheath assembly. However, we should keep in mind that in contrast to CFAP65 KO mice, TPPP2 KO mice did not display structural abnormalities.

From an evolutionary point of view, an interesting point is that the *Ch. reinhardtii* paralogs of both CFAP65 (FAP65 protein) [53] and TPPP2 (FAP265 protein) [29] are flagellar proteins and have a role in flagellar functions. The flagella of bi-flagellated alga *Ch. reinhardtii* have a similar axoneme structure to mammalian sperm flagella (nine doublet microtubules surrounding two central single microtubules (central pair complex)); thus, analogies between flagella of these various species may provide useful information. A nice example is that in *Ch. reinhardtii*, FAP65 is part of the central pair complex [53], while the sperm flagella of CFAP65 KO mice often lack the central pair complex [9], similarly to patients with CFAP65 mutations [50].

## 10. Conclusions

As can be seen, relatively few papers have been published not only on the relationship between TPPP2 and spermiogenesis, but also on TPPP2 in general. This makes it easier to summarize the results, but this way, one cannot have even an approximate complete knowledge of the topic. It is advantageous that, in addition to human data, there are also data from many other mammals. Based on the results reviewed here, it can be said that TPPP2, one of the three paralogs of vertebrate TPPP proteins, is expressed specifically in male reproductive system, mainly in the testes and sperm and also in the epididymis. In the testes, TPPP2 is exclusively expressed in elongating spermatids; in the epididymis, it is located in the middle piece of the sperm tail [8,50]. TPPP2 is involved in spermiogenesis, in the steps which are determinative for the formation and morphology of spermatids [8]. There is an accordance in the reports that there is a connection between TPPP2 levels on the one hand, and fertility and the properties of the sperm on the other hand. The over- or under-expression of this protein has functional consequences. The inhibition of TPPP2 decreases sperm motility (the curvilinear velocity of sperms [6]), probably due to influencing mitochondrial energy production since TPPP2 KO mice possess an impaired mitochondrial structure [8].

The data were obtained using various mammalian species: human, mouse, swine, and various ruminants; there is a significant homology among TPPP2s from different species (Figure S1); moreover, in some cases, the same experiments using human and mouse samples provided the same results. It seems that there is an uncertainty in some proteomic investigations when animals of high and low fertility were compared. TPPP2 was found either in the up- [3] or in the down-regulated [49] proteins in animals of low fertility. Up- and down-regulation was obtained with buffalo (*Bubalus bubalis*) and bull (*Bos taurus*), respectively, which possess identical (100%) TPPP2 (Figure S1) due to the close phylogenetic proximity (same subfamily) of the two species. Thus, it is improbable that the different results are due to the differences in species. In both studies, a relatively small sample size was used; the fertility based on the conception rates and the differences between these rates were not the same in the two studies. It cannot be excluded that both high and low expression of TPPP2 can be disadvantageous for fertility. However, from experiments with *Tppp2*^−/−^-mice, it is certain that absence of TPPP2 results in serious dysfunction of sperm and causes oligoasthenozoospermia [8]. Finally, a bioinformatic work, which analyzed testicular microbiopsy data obtained by others, found 10-fold up-regulation in samples obtained from patients with non-obstructive azoospermia [7]. It may mean a “desperate attempt” for a compensation mechanism, without success, or it simply means that a very high TPPP2 level is also harmful. Further investigations are needed to obtain answers to these open questions.

## Figures and Tables

**Table 1:** Articles/research discussed in the review.

Paper	Year	Species	Source/Object	Main Findings and Effects
Liu [1]	2013	human	testis, sperm	
Martins [2]	2013	sheep	seminal plasma	
Aslam [3]	2019	buffalo	sperm	TPPP2 is overexpressed 2.2x in low fertility sperm
Zhu [8]	2019	human, mice	sperm, testis, epididymis	sperm counts and motility of KO mice are lower; inhibition of TPPP2 decreased in vitro fertilization rate; mitochondrial dysfunction
Gódia [6]	2020	swine	sperm	high positive correlation between TPPP2 and curvilinear velocity of sperms; TPPP2 is the 5th most abundant mRNA in sperm
Wang [9]	2021	mice	testis, sperm	TPPP2 is downregulated in CFAP65 KO mice
Zhang [4]	2022	yak	sperm	upregulated in frozen-thawed high motility sperms
Alagundagi [7]	2023	human	testis	TPPP2 is upregulated 10x in NOA patients
Gacem [5]	2023	bull	sperm	TPPP2 is upregulated in high fertility bulls
Xu [10]	2023	human	testis, tumors	TPPP2 is prognostic indicator of tumor (hepatocarcinoma)

## Data Availability

Not applicable.

## Supplementary Materials

The following supporting information can be downloaded at: https://www.mdpi.com/article/10.3390/ijms25137017/s1, Figure S1: Multiple sequence alignment of TPPP2 proteins of various species mentioned in the text using Clustal Omega [54].
