# Supplementary material for: The Role of Tubulin Polymerization-Promoting Protein2 (TPPP2) in Spermatogenesis: A Narrative Review
Source: Int J Mol Sci. 2024 Jun 27;25(13):7017. doi: 10.3390/ijms25137017 (PMC11241133; doi:10.3390/ijms25137017)
Supplement: Supplementary file 1 [file ijms-25-07017-s001.zip › ijms-3054357-supplementary.pdf]

Figure S1

```
Homo        maseaektfhrfaafgesssssgtemnnknfsklckdcgimdgktvtstdvdivfskvkak     60
Mus         maseaertfhrfavfgessssskeitnknfsklckdcdimdgkavtstdvdivfskvkak     60
Sus         maseaektfqrfavfgessssgtemnnknfsklckdcgimdgkmvtstdvdivfskvkak     60
Ovis        maseaertfqrfavfgessssgtemnnknfsklckdcgimdgktvtstdvdivfskvkak     60
Bos         maseaertfqrfavfgessssgtemnnknfsklckdcgimdgktvtstdvdivfskvkak     60
Bubalus     maseaertfqrfavfgessssgtemnnknfsklckdcgimdgktvtstdvdivfskvkak     60
            ******:**:***.*******..*:.***********.***** ****************

Homo        nartitfqqfkeavkelgqkrfkgkspdevleniyglmegkdpattgatkattvgavdrl     120
Mus         nartinfqqfqeamkelgqkrfkgknpdealqgvfklmegkdpattgvtkslttvggvdrl     120
Sus         nartvtfqqfqeamkelgqkrfkgkspdealeniyklmegkdpattgvtkattvggvsrl     120
Ovis        nartitfqqfqeamkelgqkrfkgkspdealeniyklmegkdpattgvtkattvggvsrl     120
Bos         nartitfqqfqeamkelgqkrfkgkspdealeniyklmegkdpattgvtkattvggvsrl     120
Bubalus     nartitfqqfqeamkelgqkrfkgkspdealeniyklmegkdpattgvtkattvggvsrl     120
            ****:.****:**:***********.***.*:.:: ***********.**:****.*.**

Homo        tdtskytgthkerfdesgkgkgiagreemtdntgyvsgykgsgtydkktk-  170
Mus         tdtskytgthkerfdesgkgkgiegreettdnsgyvsgykgagtydkknq-  170
Sus         tdtskytgthkerfdesgkgkgiagredvtdnsgyvsgykgagtydkkgst  171
Ovis        tdtskytgthkerfdesgkgkgiagredvtdnsgyvsgykgagtydkkgnn  171
Bos         tdtskytgthkerfdesgkgkgiagredvtdnsgyvsgykgagtydkkgsn  171
Bubalus     tdtskytgthkerfdesgkgkgiagredvtdnsgyvsgykgagtydkkgsn  171
            ********************** ***: ***:********:****** .
```

Figure S1. Multiple sequence alignment of TPPP2 proteins of various species mentioned in the text using Clustal Omega [54]. Amino acids, which are identical and biochemically similar in each protein with at most one exception, are indicated by black or gray shades. Homo – *Homo sapiens* NP_776245; Mus - *Mus musculus* NP_001122106; Sus - *Sus scrofa* XP_003362045; Ovis - *Ovis aries* XP_027825885; Bos - *Bos taurus* NP_001029456; Bubalus - *Bubalus bubalis* XP_025151807.

Percent Identity Matrix

1: Homo	100.00	84.12	90.00	90.59	90.59	90.59
2: Mus	84.12	100.00	87.06	88.24	88.24	88.24
3: Sus	90.00	87.06	100.00	97.08	97.66	97.66
4: Ovis	90.59	88.24	97.08	100.00	99.42	99.42
5: Bos	90.59	88.24	97.66	99.42	100.00	100.00
6: Bubalus	90.59	88.24	97.66	99.42	100.00	100.00

Percent Similarity Matrix

1: Homo	100.00	91.76	94.15	95.32	95.32	95.32
2: Mus	91.76	100.00	91.81	92.39	92.39	92.39
3: Sus	94.15	91.81	100.00	98.24	98.83	98.83
4: Ovis	95.32	92.39	98.24	100.00	99.42	99.42
5: Bos	95.32	92.39	98.83	99.42	100.00	100.00
6: Bubalus	95.32	92.39	98.83	99.42	100.00	100.00
